# A Giant Ovarian Cyst in a Neonate with Classical 21-Hydroxylase Deficiency with Very High Testosterone Levels Demonstrating a High-Dose Hook Effect

**DOI:** 10.4274/Jcrpe.685

**Published:** 2012-09-11

**Authors:** Tülay Güran, Gözde Yeşil, Ömer Güran, Suna Cesur, Oktav Bosnalı, Ayşenur Celayir, Sevilay Topçuoğlu, Abdullah Bereket

**Affiliations:** 1 Marmara University, Pediatric Endocrinology and Diabetes, Istanbul, Turkey; 2 Zeynep Kamil Maternity and Childrens Diseases Research and Training State Hospital, Genetics, İstanbul, Turkey; 3 Şisli Etfal Research and Training State Hospital, Neonatal Intensive Care Unit, İstanbul, Turkey; 4 Zeynep Kamil Maternity and Childrens Diseases Research and Training State Hospital, Pathology, İstanbul, Turkey; 5 Zeynep Kamil Maternity and Childrens Diseases Research and Training State Hospital, Pediatric Surgery, İstanbul, Turkey; 6 Zeynep Kamil Maternity and Childrens Diseases Research and Training State Hospital, Neonatal Intensive Care Unit, İstanbul, Turkey

**Keywords:** 21-hydroxylase deficiency, Ovarian cyst, testosterone, ambiguous genitalia, hook effect, Neonate

## Abstract

Congenital adrenal hyperplasia (CAH) is a group of disorders affecting the adrenal steroid synthesis. The most common form, 21-hydroxylase deficiency (21-OHD), leads to decreased production of cortisol and aldosterone with increased androgen secretion. In classic CAH, glucocorticoid treatment can be life-saving and serves to bring the symptoms under control. However, the treatment challenge is to effectively control the excess androgen effect by using the lowest possible glucocorticoid dose. Previous studies suggested a relationship between ovarian cyst formation and adrenal androgen excess, but neonatal large ovarian cysts have been very rarely reported in newborns with CAH. Here, we present the unique case of a neonate with classical 21-OHD who underwent surgery for a giant (10x8x7 cm) unilateral solitary ovarian follicular cyst on the 2nd postnatal day. Hormonal evaluation of the patient revealed high-dose hook effect for serum testosterone levels for the first time by a two-site immunoradiometric assay. Possible mechanisms by which androgen excess may cause ovarian cyst formation are discussed.

**Conflict of interest:**None declared.

## INTRODUCTION

21-hydroxylase deficiency (21-OHD) is the most common form of congenital adrenal hyperplasia (CAH, OMIM 201910) and causes in-utero and postnatal virilisation of females due to increased adrenal androgens. While neonatal ovarian cysts have been reported to occur in association with placental dysfunction, maternal diabetes, and toxemia, only three cases of solitary neonatal ovarian cysts associated with CAH have been reported to date (1,2). 

In 21-OHD, multiple small ovarian cysts (microcysts) can develop, especially in adolescents with the nonclassical form of the disease (3). There is only one report of multiple ovarian cysts in a neonate with the classical form of 21-OHD (4), whereas unilateral solitary giant ovarian cyst has not yet been reported in these patients. 

This paper describes a neonate with classical 21-OHD who underwent surgery for a giant unilateral solitary ovarian cyst and whose hormonal evaluation revealed a high-dose hook effect for serum testosterone levels. 

## CASE REPORT

**Figure 1:**
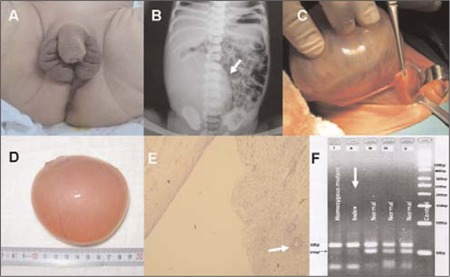
**A.** External genitalia showing severe virilization (hyperpigmentation, phallic enlargement, and labioscrotal fusion) **B.** Plain radiographic image showing a large mass deviating the intraabdominal structures **C. and D.** Gross intra-operative appearance of excised giant cyst **E.** The photomicrography showing normal epithelial lining of the simple follicular cyst. The arrow indicates a primordial follicle in normal ovarian tissue **F.** Polymerase chain reaction-restriction fragment length polymorphism (PCR-RFLP) analysis of IVS 2 mutation in the CYP21A2 gene. Digestion with Alul yielded 60bp and 34bp bands in the patient (lane II) and in homozygous mutant (positive) control (lane I), a 60 and 51bp bands in other 3 negative controls (lanes III, IV,V)

The patient presented on the first day of life with ambiguous genitalia. The pregnancy was uneventful. The family history was unremarkable with no history of consanguinity. On physical examination, length was 50 cm and body weight was 3.6 kg. The neonate had phallic enlargement, labioscrotal fusion with no palpable gonads, hyperpigmentation of external genitalia and nipples, and a large right upper quadrant abdominal mass extending into the pelvis. Abdominal and pelvic sonography revealed an infantile uterus, a large unilocular solitary right ovarian cyst measuring 10x8x7 cm (Figure 1).

The laboratory results performed on the 3^rd^ postnatal day showed elevated levels for plasma 17-hydroxyprogesterone (17OHP) (62.7 ng/mL; normal: 0.1-9.4), dehydroepiandrosterone sulphate (526 ng/mL; normal: 34-430), Δ^4^-androstenedione (10 ng/mL; normal: 0.3-3.3), adrenocorticotropic hormone (297 pg/mL; normal: 0-46) and plasma renin activity (49 ng/mL/hr; normal: 0.5-1.9). Basal and post ACTH_1-24_ stimulation test levels for 17OHP and cortisol were as follows: basal 17OHP 62.5 ng/mL, reaching 77 ng/mL, and basal cortisol 7.9 ug/dL, reaching 8.9 ug/dL (normal: 5-23 ug/dL) 60 minutes after ACTH_1-24_ administration.

Gonadotropin levels were low (luteinizing hormone <0.1 mIU/mL; normal: 2.4-12.6, follicle-stimulating hormone <0.1 mIU/mL; normal: 0.1-11). Measurement of initial serum testosterone levels yielded undetectably low values; the assay was repeated twice. This unexpected result led to the suspicion of a hook effect. To verify this suspicion, the measurement was repeated after 1/20 dilution of the serum and an excessively high level of testosterone (2739 ng/dL; normal: 6-82) was obtained. These values confirmed the diagnosis of classical salt-wasting form of 21-OHD and therapy was initiated with 15 mg/m^2^ of hydrocortisone and 0.1 mg of fludrocortisone. 

The large size of the left ovarian cyst shifting the intraabdominal structures to the left and interfering with the feeding of the baby prompted a surgical excision. The histopathologic examination revealed a follicular cyst (Figure 1). The karyotype of the baby was 46, XX. To further confirm the diagnosis of CAH, a genomic analysis was performed from the DNA of peripheral blood leucocytes and the patient was found to be homozygous for I2A/C656G mutation (intron 2 splicing mutation) in the CYP21A2 gene (5) (Figure 1). 

## DISCUSSION

Here, we present a neonate with classical salt-wasting 21-OHD due to a point mutation I2A/C656G of [i]CYP21A2[/i], which is one of the most frequent mutations in the classic 21-OHD and causes premature splicing of the intron and a shift in the translational reading frame (5). The patient presented at birth with severely virilized external genitalia and a rare finding of a solitary giant ovarian follicular cyst.

In the classical form of 21-OHD, synthesis of cortisol from cholesterol is impaired and prenatal exposure to potent androgens such as testosterone and Δ^4^-androstenedione at critical stages of sexual development virilizes the external genitalia of genetic females, resulting in genital ambiguity at birth. Newborns with salt-wasting CAH caused by 21-OHD are at risk for life-threatening salt-wasting crises. 

Abdominal cystic formations in newborns are relatively common, encountered in 1/500-1 000 live births. In female cases, the prevalence of ovarian cysts represents approximately 75% of all cysts in the abdominal cavity (6). Diagnosis is usually suspected even before birth during prenatal ultrasound scans (7). 

Functional (nonneoplastic) cysts in ovaries include follicular, corpus luteum, and theca-lutein types, all of which are benign and usually self-limited. Follicular ovarian cysts in fetuses and neonates increase in frequency with advancing gestational age and with maternal complications such as diabetes mellitus, preeclampsia, and rhesus isoimmunization (8). Functional cysts in the fetal ovary most likely result from maternal hormonal stimulation [i]in utero[/i]. 98% of cysts measuring <50 mm regress spontaneously (9). Larger and complex cysts are more likely to be nonphysiologic. 

Although maternal hormonal stimulation is thought to be the primary mechanism in neonatal follicular cysts, it is possible that highly elevated fetal androgens due to CAH, as was observed in our patient, may have been responsible for the cyst formation. Previous studies suggest that there is a causal relationship between ovarian cyst formation and adrenal androgen excess (10,11). In adolescents and young women, ovarian cyst formation is more common when there is an increased adrenal androgen concentration, as in polycystic ovary syndrome and CAH due to nonclassical 21-OHD (3,12). The nonclassical form of 21-OHD, as opposed to the classical form, is particularly associated with ovarian cysts because of the long period of nontreatment with resultant androgen excess before diagnosis. However, any hyperandrogenemic condition can theoretically cause ovarian cysts. Animal studies suggest that testosterone increases follicular FSH receptors and therefore promotes follicular growth by amplifying the FSH effect (13). According to another theory, adrenal steroid excess leads to the formation of ovarian cysts by either disrupting the cyclicity of gonadotropin release or directly affecting the ovaries (1). Both of these speculated mechanisms leading to cyst formation may have been operational in our patient. 

Wakakuri et al (14) reported a giant ovarian cyst measuring 8.5x6 cm in a poorly controlled simple virilizing 21-OHD adult that regressed with glucocorticoid replacement. Shankar et al (4) reported bilateral ovarian cysts in a 8-days-old baby with salt-losing 21-OHD with a dominant cyst measuring 7x5 cm. Bilateral ovarian cysts with a dominant cyst of 6x6x5.5 cm have also been reported in a neonate with 11β-hydroxylase deficiency (2). Due to the large size of the cysts, all these 3 cases underwent surgery. Shima et al (15) reported three cases aged 1 month, 3 months and 3 days with bilateral multicystic ovaries diagnosed as congenital lipoid adrenal hyperplasia due to mutations in the steroidogenic acute regulatory protein ([i]StAR[/i]) gene. These cases were followed conservatively on medical treatment and surgery was not required (15). To our knowledge, the cyst in our patient represents the largest unilateral solitary ovarian cyst measured to date in any reported CAH case. Surgery was necessary because the cyst, due to its size, was causing difficulty in feeding the baby and also carried the risk of torsion. 

The other interesting aspect of our case was that it demonstrated a high-dose hook effect, which is a condition by which large quantities of antigen in an immunoassay system impair antigen-antibody binding, resulting in low antigen levels in laboratory assays. This phenomenon was first described by Miles (16) with a two-site immunoradiometric assay (IRMA) for ferritin. Hook effect in measurements of GH (17), PRL (18), TSH (19), FSH, LH (20), human chorionic gonadotropin (21) and aldosterone (22) have been reported previously. To our knowledge, this is the first case where a hook effect is reported for testosterone. The hook effect gives falsely low results and may lead to misdiagnosis. 

In conclusion, our case was unique in that it presented with the largest unilateral solitary ovarian follicular cyst in a salt-losing newborn with 21-OHD, and also because it demonstrated, for the first time, presence of a hook effect caused by high serum testosterone levels.
